# The Effects of 12-Week Beta-Hydroxy-Beta-Methylbutyrate Supplementation in Patients with Liver Cirrhosis: Results from a Randomized Controlled Single-Blind Pilot Study

**DOI:** 10.3390/nu13072296

**Published:** 2021-07-02

**Authors:** Barbara Lattanzi, Angelo Bruni, Simone Di Cola, Alessio Molfino, Adriano De Santis, Maurizio Muscaritoli, Manuela Merli

**Affiliations:** 1 Gastroenterology, Department of Translational and Precision Medicine, “Sapienza” University, 00185 Rome, Italy; lattanzi.b@gmail.com (B.L.); angelo_bruni@icloud.com (A.B.); simonedicola92@hotmail.it (S.D.C.); adriano.desantis@uniroma1.it (A.D.S.); 2 Internal Medicine, Department of Translational and Precision Medicine, “Sapienza” University, 00185 Rome, Italy; alessio.molfino@uniroma1.it (A.M.); maurizio.muscaritoli@uniroma1.it (M.M.)

**Keywords:** beta-hydroxy-beta-methylbutyrate, sarcopenia, liver cirrhosis, frailty

## Abstract

Background and Aim: Sarcopenia is considered an important risk factor for morbidity and mortality in liver cirrhosis. Beta-hydroxy-beta-methylbutyrate (HMB) has the potential to increase muscle mass and performance by stimulating protein synthesis and reducing muscle catabolism. The present study aimed at evaluating the effect of HMB supplementation on muscle mass and function in patients with liver cirrhosis. Changes in frailty during the study were also estimated, and the safety of HMB supplementation was verified. Methods: This is a randomized, single-blind, placebo-controlled pilot trial. Twenty-four patients (14 HMB and 10 placebo) affected by liver cirrhosis were enrolled in the study. Each patient received dedicated counseling, which included nutrition and physical activity recommendations for chronic liver disease patients. Patients were randomized to receive 3 g/day of HMB or placebo (sorbitol powder) for 12 consecutive weeks. A diet interview, anthropometry, electrical bioimpedance analysis (BIA), quadriceps ultrasound, physical performance battery, Liver Frailty Index (LFI), and cognitive tests were completed at enrolment (T0), at 12 weeks (T1), and 24 weeks after enrolment (T2). Results: At baseline, the two groups were similar in demography, severity of liver disease, muscle mass, muscle function, and cognitive tests. LFI at baseline was higher in patients in the HMB group vs. those in the placebo group (4.1 ± 0.4 vs. 3.4 ± 0.6, *p* < 0.01). After treatment, a statistically significant increase in muscle function was seen in the HMB group (chair stand test: 14.2 ± 5 s vs. 11.7 ± 2.6 s, *p* < 0.05; six-minute walk test: 361.8 ± 68 m vs. 409.4 ± 58 m, *p* < 0.05). Quadriceps muscle mass measured by ultrasound also increased (4.9 ± 1.8 vs. 5.4 ± 1.8 mm, *p* < 0.05) after HMB, while LFI decreased (4.1 ± 0.4 vs. 3.7 ± 0.4, *p* < 0.05). HMB was well tolerated by patients, and no adverse events were documented. Conclusions: Our study suggests the efficacy of 12-week beta-hydroxy-beta-methylbutyrate supplementation in promoting improvements in muscle performance in compensated cirrhotic patients. LFI was also ameliorated. Further studies with a greater number of patients are required to reinforce this hypothesis.

## 1. Introduction

Sarcopenia represents an important burden in patients with liver cirrhosis and is associated with a high risk of morbidity and mortality [1,2,3,4,5,6]. Sarcopenia may contribute to physical frailty, which is frequently encountered in patients with advanced chronic diseases, representing an unfavorable prognostic factor for morbidity and mortality [7,8,9,10,11,12].

Given the multifactorial pathogenesis of sarcopenia and frailty, and the lack of a complete knowledge of the interactions between factors involved in their onset, a global and standardized approach for the prevention and treatment of these entities has not yet been defined. However, due to their dynamic nature and in consideration of their potential reversibility, treatments that may contribute to the management of these conditions are relevant.

Beta-hydroxy-beta-methylbutyrate (HMB) is a metabolite derived from leucine and its ketoacid alpha-ketoisocaproate (α-KIC), which has been shown to influence muscle protein turnover by stimulating protein synthesis and decreasing proteolysis [13].

Multiple pathways have been identified in which HMB plays a role in modulating skeletal muscle mass, both in vivo and in vitro [14,15]. The first mechanism is the proliferation of myoblasts through the activation of the growth hormone/insulin-like growth factor-1 (GH/IGF-1) axis, which increases the proliferation, differentiation, and survival of myoblasts, resulting in a reduction in muscle atrophy and improvements in muscle strength [15,16,17]. The role of HMB in suppressing proteolysis has also been demonstrated through the inhibition of the ubiquitin–proteasome pathway in models of neoplastic cachexia [18] as well as through the effect of attenuation of muscle atrophy secondary to steroid therapy [19]. Another pathway in which HMB plays a role is regulated by the enzyme mTOR, a stimulator of protein synthesis [13,20]. Other effects of HMB that could potentially affect muscle growth and performance include the expression of the proliferation marker MyoD and the muscle differentiation markers MRFs, as well as the stimulation of myogenic cell proliferation via the MAPK/ERK and PI3K/Akt pathways [21]. In a recent systematic review, HMB was reported to improve lean muscle mass and function in older people with sarcopenia or frailty [22].

The combination of its anabolic and anti-proteolytic properties makes HMB a potentially effective supplement for the treatment of sarcopenia in cirrhotic patients. However, there are currently no data in the literature on the use of HMB in patients with chronic liver disease. In a recent pilot study, we randomized patients after liver transplantation for supplementation with HMB (3 g/day) or placebo for 12 weeks. We observed a significant increase in appendicular lean mass (measured with DEXA) and in the hand-grip strength in patients receiving HMB supplementation versus those treated with placebo, suggesting that HMB could improve the recovery of skeletal muscle in liver transplant patients [23].

The present monocentric, randomized pilot trial was aimed at evaluating the effect of 12-week HMB supplementation vs. placebo on muscle mass and performance in a group of patients with liver cirrhosis. Secondary endpoints were the assessment of the tolerance and safety of HMB in cirrhotic patients, changes in frailty, and changes in liver function or cognitive performance during treatment.

## 2. Materials and Methods

The study was approved by the Local Ethic Committee (479/18) and registered in clinical trial.gov (NCT03892070). A randomized, controlled pilot study was planned, and patients were randomly assigned either to the treatment group (HMB 3 g/day) or the placebo group (sorbitol 3 g/day) for 12 weeks. Both groups received nutritional and physical activity counseling at the beginning of the study.

After obtaining informed consent, consecutive patients with liver cirrhosis followed by the outpatients’ clinic for “Liver cirrhosis and Portal Hypertension” at the University Hospital Policlinico Umberto I were considered for inclusion. The diagnosis of liver cirrhosis was based on liver function tests and clinical and instrumental data (ascites and/or esophageal varices and/or elastography > F4) when histology was not available. To avoid the effect of age on skeletal muscle mass, only patients <70 years of age were included in the study. Exclusion criteria were a diagnosis of hepatocellular carcinoma or other neoplastic diseases, previous liver transplantation, steroid therapy, neuromuscular or skeletal diseases, heart failure with NYHA ≥ III, severe pulmonary dysfunction, or chronic renal insufficiency stage ≥ III. Patients with active alcohol abuse in the previous six months were also excluded.

### 2.1. Protocol of the Study

All of the patients were extensively informed about the concepts of frailty and sarcopenia in chronic liver disease. The objective and methods of the study were illustrated, and all patients signed a written informed consent form before participating in the study.

Once the inclusion and exclusion criteria were verified, demographic data (including name, gender, age) as well as main anthropometric data (weight, height, BMI) were recorded. In the presence of ascites or water retention, body weight was corrected accordingly [24]. At baseline (T0), biochemical and clinical data were recorded, and MELD and Child–Pugh scores were calculated. Comorbidities (diabetes, arterial hypertension, cardiac insufficiency, etc.) and ongoing therapies were always noted. During the visit at T0, patients completed a 3-day food diary and a questionnaire to assess their physical activity. Following questionnaire compilation, each patient participated in a motivational interview where they received information and counseling on nutrition and physical activity according to European guidelines and recommendations [24]. The information provided was also delivered to the patient and the caregiver in the form of brochures specifically prepared for this category of subjects.

The enrolled patients underwent a complete physical performance battery (six-minute walk test, hand-grip test, and chair stands test), an assessment of the Liver Frailty Index, and cognitive tests (animal naming test, Mini-Mental State Examination, and psychometric hepatic encephalopathy score). For the evaluation of muscle mass, the patients underwent bioelectric impedance analysis (BIA) to measure the fat mass index, fat-free mass index, and phase angle. Ultrasound was also performed to measure the thickness of the quadriceps femoris muscle.

After randomization, the supplement to be taken during the study (treatment or placebo) was given to the patients as a white powder in sachets, with directions for it to be taken twice a day. The powder was indistinguishable for solubility and taste between treatment and placebo. Patients received periodic telephone consultations to check the progress of the study, compliance, and the occurrence of any critical issues.

After 12 weeks (T1), the patients returned to the outpatients’ clinic to repeat measurements and physical and neurocognitive tests carried out at the time of enrolment. Subsequently, the study continued with a further observation period of 12 weeks (T2) after treatment discontinuation.

### 2.2. Methods

#### 2.2.1. Bioelectric Impedance Analysis

BIA was performed to estimate body composition (BIA Inbody 770, USA). Indices of muscle mass and adiposity were estimated by using a program provided by the producer, including fat FFMI (calculated as kilograms divided by meters squared), fat mass index (FMI (calculated as kilograms divided by meters squared)), and phase angle (PhA).

#### 2.2.2. Quadriceps Ultrasound

The ultrasound measurement of the quadriceps femoral muscle was always performed by the same operator (B.L.) who was not aware of the patient’s treatment. The quadriceps thickness was measured according to a previously described method [12]. The measurement was carried out with the patient in the supine position, oriented at the center of an imaginary line between the anterior-superior iliac spine and the upper edge of the patella. Three measurements were initially made by placing the probe on the skin without pressure (thickness without pressure), and another three were made by applying pressure (thickness with pressure). The average values were divided by the body height squared to obtain the thickness without pressure index (TWI) and the thickness with pressure index (TPI).

#### 2.2.3. Liver Frailty Index

The Liver Frailty Index is a score previously validated for cirrhosis. It is derived from an algorithm that analyzes the data obtained by the balance tests, hand-grip (HG) test, and chair stand test. The score identifies three classes of patients according to the degree of frailty: Robust (LFI < 3.2), Pre-Frail (LFI 3.3 > 4.4), and Frail (LFI > 4.5) [7].

Caloric intake was evaluated through a three-day food diary and decoded using WinFood nutrition software (Medimatica, TE Teramo, Italy) according to the table of food consumption of the Italian National Institute of Nutrition [25] and Food Composition Database for Epidemiological Study in Italy [26]. For the evaluation of daily physical activity, patients completed the International Physical Activity Questionnaire (IPAQ) [27].

For the evaluation of cognitive status, the psychometric hepatic encephalopathy score and the (PHES) animal naming test (ANT) were carried out according to previous studies [28,29].

## 3. Statistical Analysis

In consideration of the lack of data on HMB supplementation in patients with liver cirrhosis, a pilot study was designed to collect data that can eventually be utilized in the design of future trials. A sample size of 14 subjects for each arm was chosen based on studies previously performed in other patient populations [30]. All clinical, laboratory, and instrumental data of the patients were collected in a pre-established database in accordance with the laws for the protection of privacy. Pearson’s chi-square test was used for the comparison of categorical variables. Student’s *t*-test was used for continuous variables. The randomization list was computer generated based on random numbers. Paired Student’s *t*-test was used to evaluate changes in muscle parameters before and after treatment or placebo. All data are expressed as mean and standard deviation or percentage when indicated. Values of *p* ≤ 0.05 were considered statistically significant. The Number Cruncher Statistical System (NCSS) 2007 statistics program was used to process the data.

## 4. Results

### 4.1. Study Population

A total of 27 patients affected by liver cirrhosis were prospectively enrolled and randomized, 14 in the HMB group and 13 in the placebo group. Three patients discontinued the study: one patient had to move outside the region for his job and asked to leave the study, and two patients discontinued the study after two weeks of enrolment due to lack of compliance. All of these patients were from the placebo group. We analyzed the results of 14 patients in the HMB and 10 patients in the placebo group. Participants’ demographic and clinical characteristics are shown in Table 1. The majority of patients were classified in Child–Pugh class A, a small number in Child–Pugh class B, and no patients in class C. Age, sex, and liver disease severity were not significantly different between the two groups at baseline. Viral etiology was more frequent in the placebo group.

### 4.2. Basal Evaluation

BMI, body composition evaluated by BIA, and muscle function were similar in the two groups. The LFI score was higher in the HMB group (*p* = 0.01) The cognitive assessment showed no significant differences. Eating habits, obtained from the food questionnaire administered at baseline, showed that the large majority of patients were consuming a low-protein (<1.2 g/kg) and a low-calorie diet (about 500 Kcal lower than what is recommended by European Guidelines daily [23]) with no differences between groups. Daily physical activity levels (assessed using the IPAQ questionnaire) were also similar between the two groups: patients were “sufficiently active” or “inactive” (Table 1), and no patients were found to be “very active”.

### 4.3. Modifications Induced by Treatment

The BMI and body composition assessed through BIA remained substantially unchanged (Table 2) during the study in both groups. Quadriceps femoral thickness measurement using ultrasound was performed at T0, T1, and T2 in all patients except one patient from the HMB group. As shown in Table 3, a significant increase in TPI at T1 vs. T0 was observed in the HMB group (4.9 ± 1.8 vs. 5.4 ± 1.8 *p* = 0.03). This increase was maintained at T2 (4.9 ± 1.8 vs. 5.4 ± 1.7 *p* = 0.03). In the placebo group, both TPI and TWI did not show significant differences (Table 3).

### 4.4. Muscle Performance Analysis

Hand-grip strength was not significantly modified during the study. However, the chair stand test improved at T1 only in the patients supplemented with HMB (from 14.2 ± 5 s to 11.7 ± 2.6 s *p* < 0.05). This improvement was maintained at T2 (Table 4). The 6-MWT also improved in the HMB group at T1 (from 361.8 ± 68 m to 409.4 ± 58 m (*p* < 0.05). This improvement was still present at T2. In the control group, these parameters showed no significant modifications. 

### 4.5. Frailty

**Figure 1:**
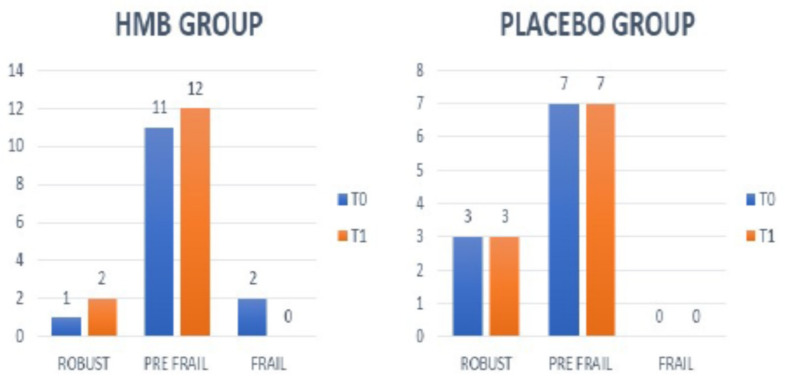
Modification of frailty during follow-up in patients of placebo and HMB group.

An improvement in frailty was observed only in the HMB group (LFI from 4.1 (±0.4 SD) at T0 to 3.7 (±0.4 SD) at T1 (*p* = 0.04)). This reduction was maintained at T2 (Table 5). In the HMB group at T0, 2 patients were frail, 11 were pre-frail, and 1 patient was robust. At the end of 12-week HMB supplementation, two frail subjects became pre-frail and one pre-frail subject became robust (Figure 1). In the control group, no significant changes were observed.

### 4.6. Liver Function, Disease Severity, and Safety of HMB

The overall severity of cirrhosis (MELD and Child–Pugh score) was stable over the observation period for both groups. Liver function test results did not change during the HMB supplementation period (Table S1). During HMB treatment, no adverse events were registered. The HMB was well tolerated by all patients.

### 4.7. Psychometric Hepatic Encephalopathy Score and Animal Naming Test

PHES and the ANT results were not significantly modified during the study in either group (Table S2).

### 4.8. Food Diary and Daily Physical Activity

The food diaries, administered at T0 and T2, were used to evaluate the adherence to nutritional advice during the study. There was a significant increase in calorie intake at the end of the study vs. the beginning of the study for both groups (1675 ± 360 vs. 1957 ± 522; *p* = 0.007). Similarly, protein intake was significantly increased (0.9 ± 0.3 vs. 1.13 ± 0.3; *p* = 0.005). Regarding daily physical activity, counseling induced a homogeneous improvement in physical activity in both groups.

## 5. Discussion

In this pilot study, we found that HMB supplementation increased the muscle performance of cirrhotic patients compared to that of the placebo group. Lean body mass as measured by BIA was not modified, but quadriceps thickness, measured using ultrasonography, was slightly but significantly improved. LFI was also ameliorated in the HMB group but not in the control group. However, this improvement should be considered with caution, as the patients in the HMB group had a worse LFI at enrolment.

A depletion in muscle mass and function is frequently reported in cirrhotic patients [6,9]. The mechanisms involved in sarcopenia in advanced liver disease have been extensively revised [31], and the need to improve this condition has been underlined by many authors [10,24]. Our pilot study, performed in patients with a diagnosis of well-compensated liver cirrhosis (all Child–Pugh A or B) suggests that active counseling for nutritional intake and moderate physical activity was able to prevent progressive muscle loss during a six-month observation period in both groups. A similar observation was reported in a study by Román et al. in 2016 [32]. Other studies found promising results regarding quality of life, physical function, and physical capacity by improving physical activity in cirrhotic patients [33]. Patients receiving HMB supplementation together with general lifestyle prescriptions achieved, in addition, a significant improvement in muscle performance. Both the distance walked by patients in the six-minute walk test (6MWT) and the time required to get up five times from the chair (CST) were significantly improved vs. baseline.

In accordance with the study by Vallejo et al. [34] on the intracellular metabolic effects of HMB in mouse models and in agreement with the meta-analysis by Holeček et al. [35] on human studies, HMB appears to act mainly on muscle function rather than on muscle mass [30,36,37].

In consideration of the increased interest and awareness of the importance of frailty in the cirrhotic patient [7,10], we also included this parameter in the results, as sarcopenia is closely related to the concept of physical frailty.

Following our results, beta-hydroxy-beta-methylbutyrate can be considered a potential therapeutic support, in addition to lifestyle modifications, to ameliorate muscle function in cirrhotic patients.

We also reported a slight increase in quadriceps muscle mass measured using ultrasound (TPI) in the HMB group. However, BIA evaluation indicated that body composition was not substantially modified. Therefore, further studies are needed to confirm whether HMB may help improve muscle mass in the long term. Even the mild improvement in frailty in the HMB group vs. controls could be promising, although the exact interpretation of this result was jeopardized by the difference in LFI in the two groups at baseline.

Regarding safety and tolerability, none of the Child–Pugh A/B cirrhotic patients showed intolerance or presented with any side effects during HMB supplementation, and liver function tests were unmodified.

In the study, we also included the analysis of the patient’s cognitive status to evaluate whether a modification in muscle mass and function could also induce changes in the cognitive state as previously reported [3,4,38,39]. However, none of the patients enrolled showed cognitive impairment at baseline, with the average PHES equal to –1 in the placebo group and −2 in the HMB group, and these parameters were unmodified during the follow-up.

The present study has some limitations: it is a pilot study that examines a limited series of cirrhotic patients. Child–Pugh C patients were not included; therefore, the efficacy and tolerability of HMB in advanced stages of the disease were not tested. Females were underrepresented, comprising only 36% of the patients. However, studies on HMB supplementation in cirrhotic patients are lacking, and the present study could stimulate larger and more prolonged protocols in the future.

In conclusion, this randomized and controlled pilot study suggests that adequate dietary intake and physical activity counseling alongside HMB supplementation for 12 weeks may improve muscle function in compensated cirrhotic patients. These positive results persisted for six months after discontinuation. Moreover, HMB supplementation is safe and well tolerated in compensated cirrhotic patients.

## Figures and Tables

**Table 1:** Demographic and clinical characteristics of the cirrhotic patients.

Variable	Placebo Group (10 Patients)	HMB Group (14 Patients)	*p* Value
Age (years)	56 ± 4.6	59.2 ± 8.4	0.3
Male gender, *n* (%)	6 (60)	9 (64.3)	0.8
Etiology of cirrhosis *n* (%)			0.05
Virus	7(70)	2 (14.2)	
Alcohol	1 (10)	4 (28.6)	
Virus + alcohol	1 (10)	4 (28.6)	
Post-Nash	1 (10)	4 (28.6)	
Esophageal varices *n* (%)			0.5
Absent	4 (40)	4 (28.6)	
F1	3 (30)	5 (35.7)	
F2 + F3	3 (30)	5 (35.7)	
Child–Pugh class *n* (%)			0.7
A	9 (90)	12 (85.8)	
B	1 (10)	2 (14.2)	
C	0	0	
MELD	9.8 ± 3.2	9 ± 2.7	0.5
PhA (°)	5.3 ± 0.5	4.9 ± 0.7	0.1
Fat mass—BIA (kg)	31.4 ± 9.9	26.4 ± 14	0.3
Lean mass—BIA (kg)	33.5 ± 7.9	31.8 ± 5.9	0.5
BMI (kg/m^2^)	29.8 ± 4.3	29.6 ± 6.8	0.9
HG (kg)	32.4 ± 12.6	26.1 ± 11.3	0.2
CST (s)	12.2 ±5.1	14.2 ± 5.1	0.3
6MWT (m)	387 ± 96	361.4 ± 68	0.4
LFI	3.4 ± 0.6	4.1 ± 0.4	0.01
PHES	−1 ± 3	−2 ± 1.5	0.3
ANT	21 ± 6.5	17 ± 6.1	0.2
Protein/kg consumption at T0 (gr/kg)	0.89 ± 0.4	0.94 ± 0.3	0.8
Calorie consumption at T0 (Kcal/24 h)	1811 ± 400	1600 ± 338	0.2
Diet regimen at T0 *n* (%)			0.5
Hypocaloric diet *n*	9 (90)	13 (93)	
Normocaloric diet *n*	1 (10)	1 (7)	
Hypercaloric diet *n*	0	0	
IPAQ questionnaire results *n* (%)			0.4
Inactive	3 (30)	5 (36)	
Sufficiently active	7 (70)	9 (64)	
Active	0	0	

Value expressed as mean ± SD. Abbreviations: BIA, bioelectric impedance analysis; BMI, body mass index; PhA, phase angle; HG, hand-grip test; CST, five-chair stand test; 6MWT, six-minute walk test; LFI, Liver Frailty Index; PHES, psychometric hepatic encephalopathy score; ANT, animal naming test; IPAQ, International Physical Activity Questionnaire Short Form.

**Table 2:** Changes in body composition according to BIA across the study.

	T0	T1	T2
HMB GROUP (14 patients)			
Lean mass/h^2^ (kg/h^2^)	11.1 ± 1.6	11.1 ± 1.7	11.2 ± 1.6
Fat mass/h^2^ (kg/h^2^)	9.3 ± 5.2	9.4 ± 5.1	9.4 ± 4.9
BMI (kg/m^2^)	29.6 ± 6.8	29.4 ± 6.8	29.2 ± 6.7
PhA (°)	4.9 ± 0.7	4.9 ± 0.8	4.8 ± 0.2
PLACEBO GROUP (10 patients)			
Lean mass/h^2^ (kg/h^2^)	11.3 ± 2.1	11.4 ± 2.1	11.4 ± 2
Fat mass/h^2^ (kg/h^2^)	10.7 ± 3.4	10.6 ± 3.8	10.3 ± 3.9
BMI (kg/m^2^)	29.9 ± 4.4	29.9 ± 4.3	29.6 ± 4.6
PhA (°)	5.3 ± 0.5	5.2 ± 0.7	5.2 ± 0.7

Value expressed as mean ± SD. Abbreviations: BIA, bioelectric impedance analysis; BMI, body mass index; °, degrees; PhA, phase angle).

**Table 3:** Changes in quadriceps thickness across the study according to ultrasounds.

	T0	T1	T2
HMB GROUP (13 patients)			
TPI (mm/h^2^)	4.9 ± 1.8	5.4 ± 1.8 *	5.4 ± 1.7 *
TWI (mm/h^2^)	10.2 ± 2.5	10.6 ± 2.3	10.7 ± 2.5
	**T0**	**T1**	**T2**
PLACEBO GROUP (11 patients)			
TPI (mm/h^2^)	5.6 ± 1.4	5.4 ± 1.1	5.4 ± 1.3
TWI (mm/h^2^)	10.2 ± 2.2	10.2 ± 2.2	10.2 ± 2.3

Value expressed as mean ± SD. * *p* ≤ 0.05 in comparison with T0. Abbreviations: TWI, thickness without pressure index; TPI, thickness pressure index.

**Table 4:** Changes in muscle performance across the study.

	T0	T1	T2
HMB GROUP (14 patients)			
HG (kg)	26 ± 11.3	28.7± 13.4	29.1 ± 13.4
FCS (s)	14.2 ± 5	11.7 ± 2.6 *	11.7 ± 2.3 *
6MWT (m)	361.8 ± 68	409.4 ± 58 *	407.3 ± 73 *
	**T0**	**T1**	**T2**
PLACEBO GROUP (10 patients)			
HG (kg)	32.4 ± 12.6	32.6 ± 12.7	33.4 ± 13.5
FST (s)	12.2 ± 5.1	11.7 ± 4.3	11.7 ± 4
6MWT (m)	387 ± 97	405 ± 72	396 ± 63

Value expressed as mean ± SD. * *p* ≤ 0.05 in comparison with T0. Abbreviations: HG, hand-grip test; CST, five-chair stand test; 6MWT, six-minute walk test.

**Table 5:** Changes in Liver Frailty Index across the study.

	T0	T1	T2
HMB GROUP (14 patients)			
Liver Frailty Index	4.1 ± 0.4	3.7 ± 0.4 *	3.6 ± 0.6 *
	**T0**	**T1**	**T2**
PLACEBO GROUP (10 patients)			
Liver Frailty Index	3.5 ± 0.6	3.3 ± 0.6	3.3 ± 0.6

Value expressed as mean ± SD. * *p* ≤ 0.05 in comparison with T0.

## Data Availability

Not applicable.

## Supplementary Materials

The following are available online at https://www.mdpi.com/article/10.3390/nu13072296/s1, Table S1: Modifications of liver tests during the study. Table S2: Modifications of cognitive test during the study.
