# Supplementary material for: The Effects of 12-Week Beta-Hydroxy-Beta-Methylbutyrate Supplementation in Patients with Liver Cirrhosis: Results from a Randomized Controlled Single-Blind Pilot Study
Source: Nutrients. 2021 Jul 2;13(7):2296. doi: 10.3390/nu13072296 (PMC8308449; doi:10.3390/nu13072296)
Supplement: Supplementary file 1 [file nutrients-13-02296-s001.zip › nutrients-1241097-supplementary.pdf]

Supplementary Materials

Table S1. Modifications of liver tests during the study.

	T0	T1	*P value*
	HMB GROUP (14 patients)		
AST (U/L)	40.55 ± 26	34.11 ± 23	N.S.
ALT (U/L)	33.6 ± 20	30.11 ± 21	N.S.
Gamma GT (U/L)	238.37 ± 311	200 ± 286	N.S.
Total Bilirubin (mg/dl)	1.4 ± 0.3	1.8 ± 0.17	N.S.
Direct Bilirubin (mg/dl)	0.5 ± 0.3	0.4 ± 0.2	N.S.
MELD	9 ± 2.7	8.5 ± 2.4	9.2 ± 4.6
CHILD-PUG SCORE	5.6 ± 0.8	5.6 ± 0.6	5.5 ± 0.7
	**T0**	**T1**	***P value***
	PLACEBO GROUP (10 patients)		
AST (U/L)	40 ± 24	38 ± 16	N.S.
ALT (U/L)	36.7 ± 11	40 ± 10	N.S.
Gamma GT (U/L)	148 ± 179	105 ± 88	N.S.
Total Bilirubin (mg/dl)	1.13 ± 0.6	1.4 ± 0..8	N.S.
Direct Bilirubin (mg/dl)	0.4 ± 0.2	0.5 ± 0.3	N.S.
MELD	9.8 ± 3.2	9.9 ± 2.6	9,9 ±2,5
CHILD-PUG SCORE	5.6 ± 0.9	5.6 ± 0.7	5.6 ± 0.7

Value expressed as mean ± SD. Abbreviations: AST (aspartate aminotransferase), ALT (Alanine Aminotrasferase), Gamma GT (Gamma Glutamyl Transferase).

Table S2. Modifications of cognitive test during the study.

	T0	T1	T2
	HMB GROUP (14 patients)		
PHES	-2 ± 1.5	**-2.3 ± 2.3**	-2 ± 1.6
ANT	17 ± 6	19 ± 4	20 ± 4
	**T0**	**T1**	**T2**
	PLACEBO GROUP (10 patients)		
PHES	-1 ± 3	-0.4 ± 2.1	0 ± 0.8
ANT	21 ± 6	21 ± 4	19 ± 5

Value expressed as mean ± SD. Abbreviations: PHES (Psychometric Hepatic Encephalopathy Score), ANT (Animal Naming Test).
